# Bridging via extracorporeal cardiopulmonary resuscitation for hemodynamic collapse in a patient with metastatic lung cancer: a case report

**DOI:** 10.1186/s40981-025-00839-z

**Published:** 2025-12-05

**Authors:** Takayuki Hasegawa, Shoe Kobiyama, Ryosuke Sasaki, Tatsusmi Yakushiji, Keisuke Yoshida, Takahiro Hakozaki, Satoki Inoue

**Affiliations:** 1 Division of Anesthesia and Pain Medicine, Fukushima Medical University Hospital, 1 Hikarigaoka, Fukushima City, Fukushima 960-1295 Japan; 2 Department of Anesthesiology, Ohta-Nishinouchi Hospital, Fukushima, Japan

**Keywords:** Epidermal growth factor receptor, Extracorporeal cardiopulmonary resuscitation, Pulmonary tumor thrombotic microangiopathy, Osimertinib, Veno-arterial extracorporeal membrane oxygenation

## Abstract

**Background:**

Due to the highly invasive nature of veno-arterial extracorporeal membrane oxygenation (VA-ECMO), advanced malignancy is considered a relative contraindication. We here report a patient with hemodynamic collapse secondary to metastatic lung cancer in whom bridging via VA-ECMO was successful.

**Case presentation:**

A 35-year-old man with metastatic non-small cell lung cancer harboring a deletion in exon 19 of epidermal growth factor receptor developed acute right ventricular failure and hemodynamic collapse due to pulmonary tumor thrombotic microangiopathy. Because treatment with the targeted agent osimertinib had already been initiated and a rapid response was anticipated, VA-ECMO was instituted as a bridge to its therapeutic effect. The patient's hemodynamics stabilized within 7 days, permitting VA-ECMO decannulation. At the time of writing, the patient is continuing to undergo regular outpatient follow-up.

**Conclusions:**

In carefully selected oncology patients with highly treatment-sensitive disease, short-term VA-ECMO may be an effective bridge to systemic therapy.

## Background

Extracorporeal cardiopulmonary resuscitation (ECPR) is a contemporary resuscitation approach that employs veno-arterial extracorporeal membrane oxygenation (VA-ECMO) [1]. Strict patient selection is essential due to the highly invasive nature of the procedure and its associated serious complications [2]. Terminal malignancy is considered a relative contraindication to ECMO, with treatment guidelines emphasizing prognosis and the likelihood of short-term reversibility when weighing harm versus benefit [3]. ECPR is often performed in emergency clinical situations, where the selection of appropriate treatment indications remains a challenge.

Among cancer-related causes of acute right ventricular failure, pulmonary tumor thrombotic microangiopathy (PTTM) is rare. Tumor cell microemboli lodge in small- to medium-sized pulmonary arteries, inducing fibrocellular intimal proliferation and vascular remodeling, which rapidly lead to severe pulmonary hypertension and right ventricular failure [4]. The natural history is fulminant, with a mean survival of 9.5 weeks from symptom onset (median 3 weeks; range < 0.5–88 weeks) and multiple reports of sudden death [5–7].

We here report a case of metastatic epidermal growth factor receptor (EGFR) mutation-positive non-small cell lung cancer (NSCLC) complicated by PTTM, which progressed to acute right ventricular failure and hemodynamic collapse. Osimertinib therapy was continued under VA-ECMO because this treatment was expected to exhibit rapid efficacy. The patient was decannulated from VA-ECMO and achieved prolonged survival.

## Case presentation

A 35-year-old man (height, 176 cm; weight, 70 kg) presented to a local clinic with fever, cough, and fatigue, and was subsequently referred to our hospital. His medical history was unremarkable except for insomnia, and his oral medications included bromazepam and lorazepam. Chest radiography and computed tomography suggested a malignant tumor, and endobronchial ultrasound-guided transbronchial needle aspiration confirmed the diagnosis of lung adenocarcinoma. Metastases to the brain, liver, and bone, and malignant pericardial effusion were also observed. In addition, genetic testing revealed a deletion mutation in exon 19 of EGFR. As such tumors often respond rapidly to osimertinib, a third-generation EGFR tyrosine kinase inhibitor [8], osimertinib treatment was initiated.

**Fig. 1:**
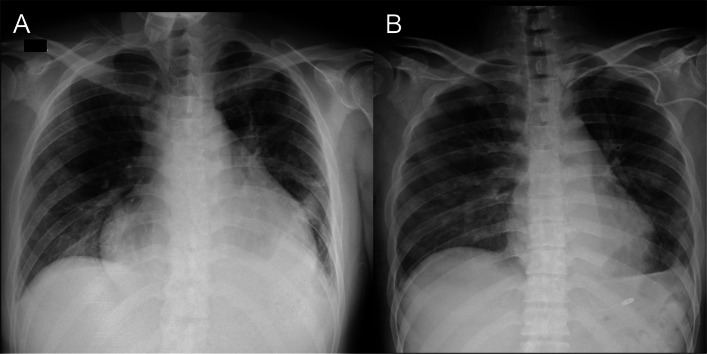
Chest radiography. **A** Chest radiography on the day of respiratory deterioration, demonstrating marked cardiomegaly. **B** Chest radiography on the day after decannulation from ECMO and removal of mechanical ventilation, revealing improvement in the cardiomegaly

**Fig. 2:**
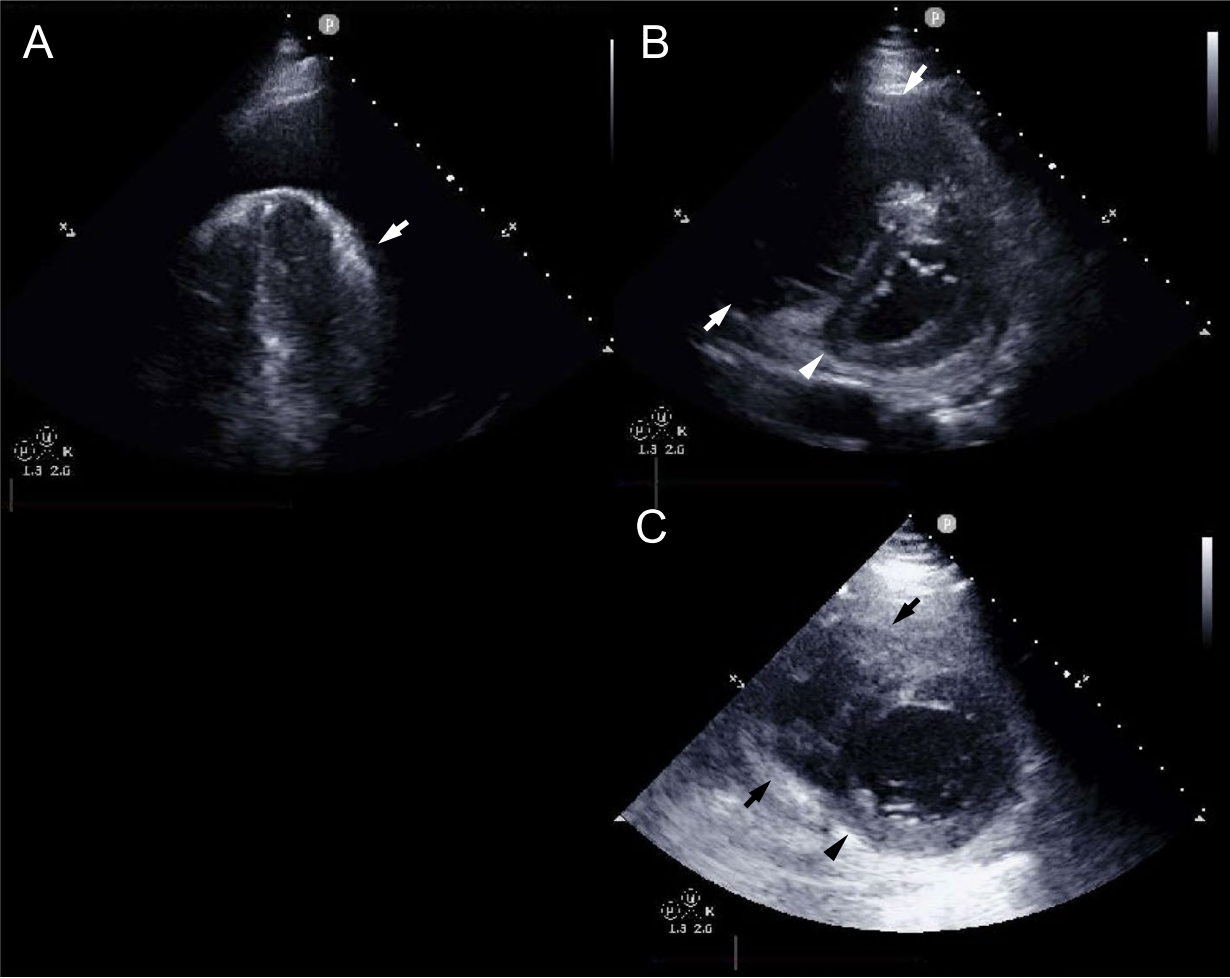
Transthoracic echocardiography (TTE) images. **A** Apical view obtained before pericardial drainage. The white arrow indicates the heart and the surrounding echo-free space represents pericardial effusion. **B** Left ventricular short-axis view at end-diastole after pericardial drainage. The pericardial effusion had resolved; however, the right ventricle became dilated (white arrows) and the left ventricle was deformed into a D-shape (white arrowhead). **C** Left ventricular short-axis view obtained immediately prior to decannulation, with the ECMO flow reduced to 1.5 L/min. The right ventricular size had returned to normal (black arrows). The D-shaped deformation of the left ventricle also improved and the ventricle showed a nearly circular shape (black arrowhead)

**Fig. 3:**
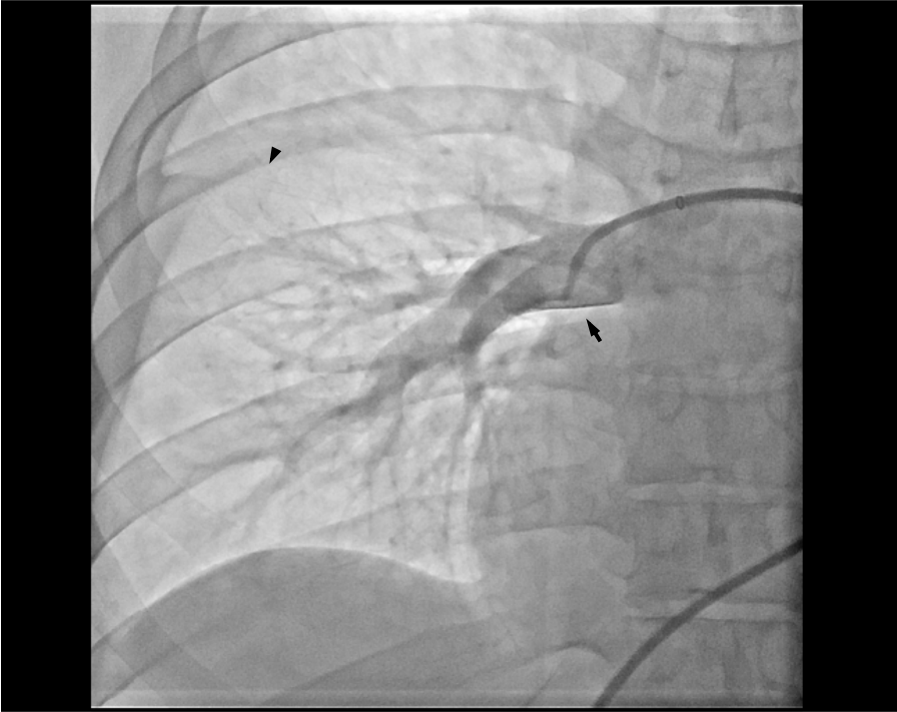
Representative pulmonary angiography of the right lower lobe. No massive thrombus was observed in the main pulmonary arteries (black arrow), excluding hemodynamic instability due to pulmonary embolism. The peripheral pulmonary arteries were opaque with delayed filling (black arrowhead), suggesting PTTM. Similar findings were observed in the other pulmonary arteries

On the first day of osimertinib treatment, the patient developed dyspnea and orthopnea. No pulmonary opacities were noted on chest radiography, but cardiomegaly was observed (Fig. 1A). Transthoracic echocardiography (TTE) demonstrated large pericardial effusion (Fig. 2A), suggesting cardiac tamponade as the cause of the respiratory symptoms. Therefore, pericardial drainage was performed, removing 1000 mL of hemorrhagic fluid, and follow-up TTE confirmed drainage of the pericardial effusion. However, the tricuspid regurgitation pressure gradient remained high at 33.8 mmHg, tricuspid annular plane systolic excursion decreased to 12 mm, and a D-shaped left ventricle was observed (Fig. 2B), indicating right ventricular dysfunction. Moreover, persistent dyspnea necessitated non-invasive positive pressure ventilation despite effective drainage. With a continuous positive airway pressure of 7 cm H_2_O and inspired oxygen fraction (FiO_2_) of 0.7, the peripheral oxygen saturation (SpO_2_), arterial partial pressure of oxygen (PaO_2_), and arterial partial pressure of carbon dioxide (PaCO_2_) were 99%, 145 mmHg, and 33.6 mmHg, respectively. The patient was admitted to the intensive care unit (ICU) for continuous respiratory support, and his circulatory status subsequently deteriorated. His blood pressure decreased from 97/74 mmHg without a vasoactive agent to 90/60 mmHg while receiving noradrenaline at 0.12 µg/kg/min, vasopressin at 2 U/hr, and dobutamine at 2 µg/kg/min. Based on the diagnosis of deep vein thrombosis, pulmonary embolism and right ventricular dysfunction were suspected, and pulmonary artery catheterization was performed. The pulmonary artery pressure was markedly high at 71/31 mmHg, and right atrial pressure also increased to 20 mmHg; however, the pulmonary artery wedge pressure remained within the normal range at 13 mmHg, suggesting precapillary pulmonary hypertension. Pulmonary angiography revealed peripheral microemboli in the pulmonary arteries, but there was no evidence of massive pulmonary embolism to explain the respiratory and circulatory failure (Fig. 3). Therefore, PTTM was clinically suspected.

Considering the deteriorating clinical course, a multidisciplinary medical team comprising intensivists, oncologists, nurses, and clinical engineers discussed the management plan. Palliative care is considered appropriate in the case of poor prognosis or survival. In this case however, given the EGFR exon 19 deletion mutation and expected rapid response to osimertinib [8], aggressive circulatory support, including VA-ECMO, was deemed useful as a bridge to therapeutic effect. The advanced care plan was re-evaluated, and the patient was confirmed to have a thorough understanding of his prognosis as well as the likelihood of a good response to osimertinib. He agreed to aggressive treatment, including VA-ECMO, but did not want life-prolonging treatment without meaningful benefit.

On the third night in the ICU, the continuous infusion rate of noradrenaline to maintain a mean blood pressure above 65 mmHg was increased from 0.05 to 0.2 μg/kg/min, dyspnea worsened and his arterial blood pressure decreased from 87/54 mmHg to 50/32 mmHg, without a carotid pulse. He simultaneously lost consciousness and the ability to breathe spontaneously, and cardiopulmonary resuscitation was performed according to the American Heart Association Basic Life Support guidelines [9]. Chest compressions and intravenous adrenaline (1 mg every 4 min) were immediately initiated, followed by endotracheal intubation with an 8.0-mm internal diameter tube. Arterial blood pressure and carotid pulsation transiently improved, but circulation soon stopped. As conventional resuscitation was insufficient, ECPR was initiated. 24 Fr venous and 20 Fr arterial cannulas were inserted into the right femoral vein and artery, respectively. A distal perfusion cannula was not used. VA-ECMO (HCS-CEP2, MERA Co., Ltd., Tokyo, Japan) was established approximately 40 min after the start of resuscitation, with a blood flow at 4.5 L/min during ongoing chest compressions and adrenaline administration. Following VA-ECMO initiation, the mean arterial pressure was maintained at 65 mmHg and the heart rate remained around 90 bpm with a continuous noradrenaline infusion at 0.05 μg/kg/min. Continuous vasopressin administration was discontinued. Arterial oxygen saturation (SaO_2_), PaO_2_, and PaCO_2_ from the right radial artery were 96.5%, 80 mmHg, and 33.7 mmHg, respectively.

Seven hours after VA-ECMO initiation, the patient was awake, and was able to make eye contact and follow commands while on mechanical ventilation. Therefore, a continuous infusion of morphine at 2 mg/hr with patient-control analgesia (2 mg per bolus) and remifentanil at 0.1 μg/kg/min were administered for analgesia and dyspnea. Dexmedetomidine at 0.4–0.8 μg/kg/hr was administered for light sedation, which was successfully achieved. His spontaneous breathing pattern was stable under pressure support ventilation without tachypnea or excessive effort, and muscle relaxants were not required. The circulatory status stabilized on VA-ECMO a continuous noradrenaline infusion at 0–0.05 μg/kg/min. Lactate levels increased to 9.3 mmol/L immediately after resuscitation, but rapidly decreased and remained around 1 mmol/L thereafter.

At the initiation of VA-ECMO, a flow of 4.5 L/min was required to maintain a mean arterial pressure of above 65 mmHg. As the tumor responded to osimertinib and his condition subsequently improved, the VA-ECMO flow was gradually reduced to 1.5 L/min, with improvement of the D-shaped left ventricle (Fig. 2C). On the sixth day after VA ECMO initiation, a weaning trial was initiated. The VA-ECMO flow was reduced to 1.0 L/min, and mechanical ventilation was set to pressure support mode with 5 cmH_2_O of positive end-expiratory pressure and 5 cmH_2_O of pressure support. Under these conditions, the patient's arterial blood pressure was 110/72 mmHg and his heart rate was 95 bpm with a continuous infusion of dobutamine at 2 μg/kg/min only. In addition, SaO_2_, PaO_2,_ and PaCO_2_ measured from the right radial artery were 98.0%, 101 mmHg, and 34.2 mmHg, respectively. The patient was weaned from VA-ECMO and extubated on the same day, and VA-ECMO decannulation was carried out on the seventh day after VA ECMO initiation. After decannulation, the blood pressure, heart rate, and respiratory rate were 109/55 mmHg, 117 bpm, and 23 breaths per minute, respectively, while receiving 3 L of oxygen via nasal cannula. The SaO_2_, PaO_2,_ and PaCO_2_ were 95.1%, 96.1 mmHg, and 31.2 mmHg, respectively. No vasoconstrictions were administered and only a continuous infusion of dobutamine at 2 μg/kg/min was continued. Chest radiography demonstrated improved cardiomegaly (Fig. 1B).

Although the patient exhibited no apparent cognitive impairment, spasticity of both lower limbs and blurred vision in both eyes persisted, and contrast-enhanced brain magnetic resonance imaging revealed high-intensity areas in the right parietal to occipital lobes and bilateral basal ganglia, suggesting hypoxic injury associated with resuscitation. No adverse events associated with osimertinib treatment were observed. At seven months after VA-ECMO weaning, the patient remains wheelchair-dependent but continues outpatient follow-up.

## Discussion

We report the case of a patient with hemodynamic collapse caused by PTTM in the setting of advanced lung cancer. Although VA-ECMO is generally considered a relative contraindication in patients with end-stage malignancy, the tumor in this case harbored a deletion mutation in exon 19 of EGFR, which is known to respond rapidly to the molecularly targeted agent osimertinib. ECPR was therefore implemented, which contributed to long-term survival.

Osimertinib, a third-generation EGFR tyrosine kinase inhibitor, is associated with favorable long-term survival in EGFR mutation-positive NSCLC, even in patients with advanced disease and poor overall condition, as in the present case. The prevalence of EGFR mutation-positive NSCLC is higher among Asian populations, with a reported rate of 49.1% [10]. Exon 19 deletions and L858R substitutions comprise the majority of EGFR mutations, and a previous meta-analysis reported exon 19 deletions to be associated with superior progression-free survival, overall survival, and response rates compared with L858R mutations [11]. First-line treatment with osimertinib for advanced NSCLC harboring EGFR mutations led to a median overall survival of 38.6 months [12], supporting the potential for long-term survival. In the present case, the patient was young and had no significant medical history, and his tumor harbored an EGFR exon 19 deletion mutation. As such, the provision of temporary circulatory support via VA-ECMO allowed sufficient time for osimertinib to exert its therapeutic effects, leading to a favorable outcome.

Although hemodynamic collapse due to PTTM is generally associated with a poor prognosis, appropriately selected VA-ECMO support may improve survival and enable long-term favorable outcomes. PTTM, first described by von Herbay et al. [4], is characterized by tumor-related microemboli and fibrocellular intimal proliferation. In the present case, acute pulmonary embolism was initially suspected because of the presence of deep vein thrombosis; however, it was excluded based on pulmonary angiography, and a clinical diagnosis of PTTM was established. Osimertinib treatment likely led to a rapid reduction in tumor-related microembolic burden, which reduced the right ventricular afterload associated with PTTM. This resulted in improvements in the D-shaped left ventricle and ultimately facilitated hemodynamic recovery and weaning from VA-ECMO.

In general, PTTM is associated with a poor prognosis and survival [5–7]. Godbole et al. systematically reviewed published cases and highlighted that premortem diagnosis is rare and survival is typically around 9.5 weeks [5]. Kim et al. reported a series of 10 patients with suspected PTTM in whom death occurred a median of 49 days after symptom onset [6]. Chen described a patient with lung adenocarcinoma with PTTM who died within 3 days despite supportive care because no specific treatment was recommended due to rapid clinical deterioration and poor general condition [7]. Although PTTM is not considered a treatable condition, the present case demonstrates that long-term survival is possible.

The diagnosis of PTTM is often established pathologically, with autopsy and histopathological confirmation being the gold standard [4]. However, premortem diagnosis is difficult, and most cases are only clinically suspected [5]. Pathological diagnosis was not performed in the present case, being a limitation, but we consider it a reasonable clinical diagnosis based on the pulmonary angiography findings and the patient’s response to osimertinib.

The use of ECMO warrants careful consideration in oncology patients because they carry risk factors that increase the morbidity of ECMO-related complications [13], such as hemorrhagic, thrombotic, and infectious events, which are relatively common [2]. Patients with malignancies are especially susceptible to such complications [14–16]. Moreover ECMO is theorized to further the spread of malignant cells, with some citing this as a reason to avoid its use despite being purely speculative [17].

Even in patients with systemic metastases, long-term survival may be achievable in certain malignancies, as demonstrated in this case. When managing critically ill patients with malignancy, multidisciplinary discussion regarding treatment strategy is essential, taking into account both oncological curability and the anticipated speed of therapeutic response. When appropriate, the prompt initiation of ECMO may improve long-term survival.

To uniformly consider malignancy as an absolute contraindication for ECMO may be detrimental, because favorable outcomes are possible in certain cases with high treatment sensitivity even if malignancy is advanced. The present case demonstrates that in EGFR mutation-positive NSCLC patients with acute right heart failure or hemodynamic collapse due to PTTM, VA-ECMO can serve as an effective bridge therapy when combined with molecular targeted treatment.

## Data Availability

Data sharing is not applicable to this article as no datasets were generated or analyzed for the report.
